# Elevated Cerebrospinal Fluid Anti-CD4 Autoantibody Levels in HIV Associate with Neuroinflammation

**DOI:** 10.1128/spectrum.01975-21

**Published:** 2022-01-05

**Authors:** Da Cheng, Zhenwu Luo, Xiaoyu Fu, Sophie Stephenson, Clara Di Germanio, Philip J. Norris, Dietmar Fuchs, Lishomwa C. Ndhlovu, Quan-zhen Li, Henrik Zetterberg, Magnus Gisslen, Richard W. Price, Shifang Peng, Wei Jiang

**Affiliations:** a Department of Microbiology and Immunology, Medical University of South Carolina, Charleston, South Carolina, USA; b Department of Infectious Disease, Xiangya Hospital, Central South University, Changsha, China; c Department of Neurology, University of California San Francisco, San Francisco General Hospital, San Francisco, California, USA; d Vitalant Research Institute, San Francisco, California, USA; e Department of Laboratory Medicine, University of California, San Francisco, California, USA; f Institut für Biologische Chemie, Biozentrum, Medizinische Universität Innsbruck, Innrain, Innsbruck, Austria; g Department of Medicine, Division of Infectious Diseases, Weill Cornell Medicine, New York, New York, USA; h Department of Immunology and Internal Medicine, University of Texas Southwestern Medical Center, Dallas, Texas, USA; i Department of Psychiatry and Neurochemistry, Institute of Neuroscience and Physiology, the Sahlgrenska Academy at the University of Gothenburg, Mölndal, Sweden; j Clinical Neurochemistry Laboratory, Sahlgrenska University Hospital, Mölndal, Sweden; k Department of Neurodegenerative Disease, UCL Institute of Neurology, Queen Square, London, United Kingdom; l UK Dementia Research Institute at UCL, London, United Kingdom; m Hong Kong Center for Neurodegenerative Diseases, Hong Kong, China; n Department of Infectious Diseases, Institute of Biomedicine, Sahlgrenska Academy, University of Gothenburg, Gothenburg, Sweden; o Region Västra Götaland, Sahlgrenska University Hospital, Department of Infectious Diseases, Gothenburg, Sweden; p National Clinical Research Center for Geriatric Disorders, Xiangya Hospital Central South University, Changsha, China; q Division of Infectious Diseases, Department of Medicine, Medical University of South Carolina, Charleston, South Carolina, USA; National Institutes of Health

**Keywords:** HIV-1 infection, neuroinflammation, anti-CD4 IgG, cerebrospinal fluid, HIV, anti-CD4 autoantibody

## Abstract

The mechanisms of persistent central nervous system (CNS) inflammation in people with HIV (PWH) despite effective antiretroviral therapy (ART) are not fully understood. We have recently shown that plasma anti-CD4 IgGs contribute to poor CD4^+^ T cell recovery during suppressive ART via antibody-mediated cytotoxicity (ADCC) against CD4^+^ T cells, and that plasma anti-CD4 IgG levels are associated with worse cognitive performance and specific brain area atrophy. However, the role of anti-CD4 IgGs in neuroinflammation remains unclear. In the current study, plasma and cerebrospinal fluid (CSF) samples from 31 ART-naive and 26 treated, virologically suppressed PWH, along with 16 HIV-seronegative controls, were evaluated for CSF levels of anti-CD4 IgG, white blood cell (WBC) counts, soluble biomarkers of neuroinflammation, and neurofilament light chain (NfL). We found that 37% of the PWH exhibited elevated CSF anti-CD4 IgG levels, but few or none of the PWH were observed with elevated CSF anti-CD4 IgM, anti-CD8 IgG, or anti-double-strand DNA IgG. CSF anti-CD4 IgG levels in PWH were directly correlated with neuroinflammation (WBC counts, neopterin, and markers of myeloid cell activation), but not with CSF NfL levels. Using cells from one immune nonresponder to ART, we generated a pathogenic anti-CD4 monoclonal IgG (JF19) presenting with ADCC activity; JF19 induced the production of soluble CD14 (sCD14) and interleukin-8 (IL-8) in human primary monocyte-derived macrophages via CD4 binding *in vitro*. This study demonstrates for the first time that elevated CSF anti-CD4 IgG levels present in a subgroup of PWH which may play a role in neuroinflammation in HIV.

**IMPORTANCE** This study reports that an autoantibody presents in the CNS of HIV patients and that its levels in the CSF correlate with some markers of neuroinflammation.

## INTRODUCTION

Although antiretroviral therapy (ART) has greatly reduced morbidity and mortality, the chronic central nervous system (CNS) immune response and persistent neuroinflammation remain common in people with HIV (PWH) (1, 2). The neuroinflammation in PWH is characterized by the activation of CNS myeloid cells, increased infiltrating white blood cells (e.g., myeloid cells, CD4^+^ T cells), and increased CNS proinflammatory cytokines and chemokines (3, 4). Persistent neuroinflammation is associated with cognitive decline and pathological changes in specific brain area in PWH (2), which may be a major contributing factor for HIV-associated neurocognitive disorders (HAND) and neurodegeneration diseases (4).

We previous reveal that plasma anti-CD4 IgG plays an important role in blunted CD4^+^ T cell reconstitution despite effective ART with viral suppression (5). Our recent studies identified high plasma anti-CD4 IgG levels in a cohort of PWH on suppressive ART were associated with worse cognitive performance and brain atrophy in select regions (6). The autoreactive anti-CD4 IgG induced CD4^+^ T cell death through antibody-dependent cell-mediated cytotoxicity (ADCC) (5). However, whether anti-CD4 IgG presents in the cerebrospinal fluid (CSF) and the role of anti-CD4 IgG in neuroinflammation remain unknown.

In the current project, we conducted a cross-sectional study measuring CSF levels of anti-CD4 IgG in parallel with plasma to determine if anti-CD4 autoantibodies are detectable in CSF and if they are associated with neuroinflammation and neural injury. We found that 37% of PWH presented elevated CSF anti-CD4 IgG levels despite of ART, which were directly correlated with markers of neuroinflammation and the degree of blood-brain barrier (BBB) permeability. A monoclonal anti-CD4 IgG induced soluble CD14 (sCD14) and interleukin-8 (IL-8) production in human primary monocyte-derived macrophages *in vitro*.

## RESULTS

### Elevated CSF anti-CD4 IgG levels were detected in a subgroup of HIV+ subjects.

**FIG 1:**
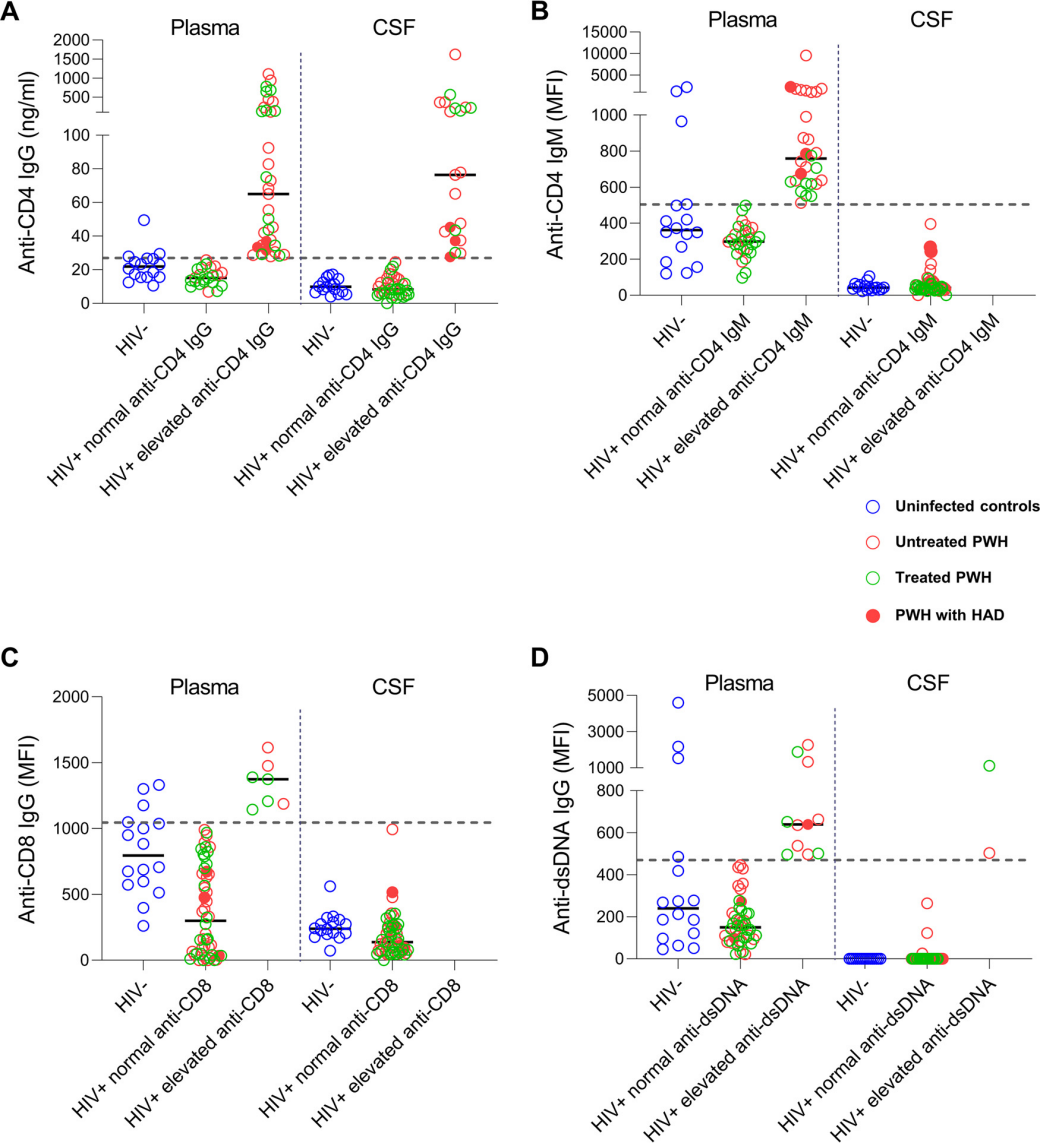
Elevated CSF anti-CD4 IgG levels in a subgroup of PWH. Elevated CSF levels of anti-CD4 IgG were found in a subgroup of PWH (panel A) but were not found in CSF anti-CD4 IgM (panel B), anti-CD8 IgG (panel C), and anti-dsDNA IgG (panel D) of PWH. Three PWH with dementia are shown as filled red circles. Blue, red, and green circles represent uninfected controls, untreated PWH, and treated PWH, respectively. The cutoff values for autoantibody levels were based on upper quartiles of corresponding plasma levels in the uninfected controls.

Recently, plasma anti-CD4 IgG levels were found to be inversely correlated with neurocognition and specific area of brain atrophy in treated HIV (6). To investigate the role of anti-CD4 IgGs in neuro-HIV pathogenesis, we analyzed anti-CD4 IgGs in CSF and paired plasma samples. Notably, 37% of the people with HIV (PWH) presented with elevated CSF anti-CD4 IgGs, and 60% of them showed elevated plasma anti-CD4 IgGs (≥27 ng/mL) (Fig. 1A). Furthermore, plasma and cerebrospinal fluid (CSF) anti-CD4 IgG levels of three HIV-associated dementia (HAD) patients were within the elevated CSF anti-CD4 IgG group (Fig. 1A).

To investigate whether CSF anti-CD4 IgG is increased specifically in some PWH, we have evaluated anti-CD8 IgG (a control autoantibody against a membrane self-antigen), anti-CD4 IgM (a control autoantibody with different isotype), and anti-double-strand DNA (anti-dsDNA) IgG (a control autoantibody against an intracellular self-antigen) as controls. The PWH were stratified into normal and elevated level subgroups for anti-CD8 IgG, anti-CD4 IgM, and anti-dsDNA IgG in both plasma and CSF samples. The cutoff values were selected based on the upper quartile of corresponding plasma antibody levels in uninfected control individuals, and the same was done for the anti-CD4 IgG grouping. Intriguingly, the CSF anti-CD4 IgM (Fig. 1B), anti-CD8 IgG (Fig. 1C), and anti-dsDNA IgG (Fig. 1D) levels were significantly lower compared with the corresponding plasma levels in PWH, while the anti-CD4 IgG levels were comparable between CSF and plasma samples of PWH (Fig. 1A). Notably, there were few or no individuals in the elevated antibody subgroups for all control autoantibodies (Fig. 1). Moreover, the ratios of anti-CD4 IgG (Fig. S1A), anti-CD4 IgM (Fig. S1B), anti-CD8 IgG (Fig. S1C), and anti-dsDNA IgG (Fig. S1D) in CSF versus plasma were not correlated with the degree of BBB permeability. These results suggest that the CSF levels of anti-CD4 IgG may be specifically elevated in a subgroup of PWH.

### Correlations between CSF anti-CD4 IgG levels and CSF WBC counts and BBB permeability.

**FIG 2:**
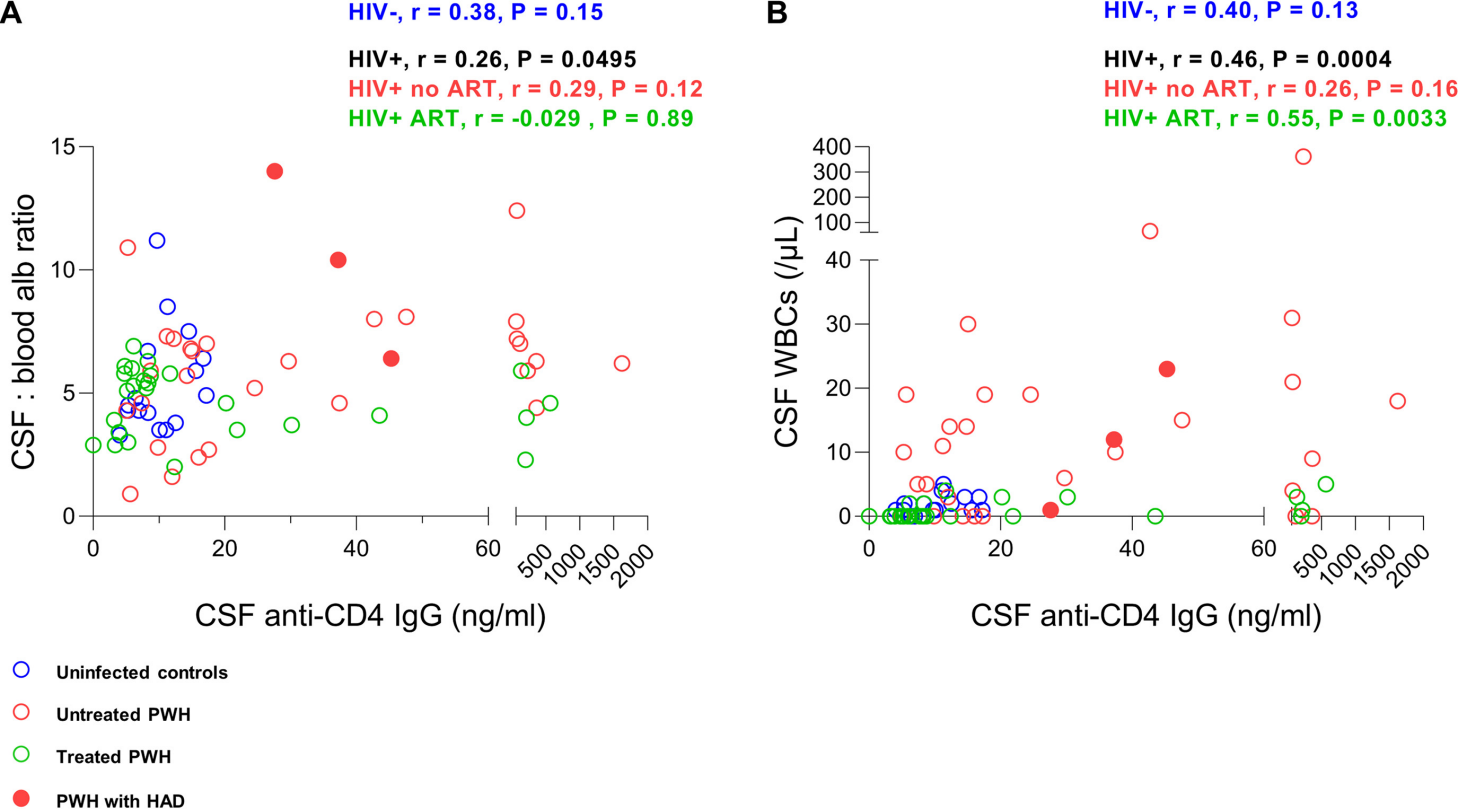
Correlations between CSF anti-CD4 IgG levels and CNS dysfunction in HIV. Correlations between CSF anti-CD4 IgG levels and the ratio of albumin in CSF versus serum (panel A) and CSF WBC counts (panel B). Three PWH with HAD are marked as filled red circles. Blue, red, and green circles represent uninfected controls, untreated PWH, and treated PWH, respectively. Spearman correlation tests were performed.

To elucidate the link between CSF anti-CD4 IgG and central nervous system (CNS) abnormalities in PWH, we evaluated the correlations between CSF anti-CD4 IgG levels and a series of markers that reflect neuroinflammation. Of note, the CSF white blood cell (WBC) counts reflecting neuroinflammation were increased in untreated PWH compared to treated PWH but were comparable between treated PWH and the healthy controls (Table 1). We found that the CSF anti-CD4 IgG levels were positively correlated with CSF WBC counts in all HIV+ subjects (*r* = 0.46, *P* = 0.0004) and treated PWH (*r* = 0.55, *P* = 0.0033), but not in antiretroviral therapy (ART)-naive PWH or in the healthy controls (Fig. 2B). The albumin ratio in CSF versus serum and CSF neurofilament light chain (NfL) indicated the magnitude of BBB permeability and neuronal injury, respectively (7, 8). Consistently, we found a weak correlation between CSF anti-CD4 IgG levels and the albumin ratio of CSF versus serum in all HIV+ subjects (*r* = 0.26, *P* = 0.0495) but not in any single HIV study group or in the healthy controls (Fig. 2A). Moreover, the degree of BBB permeability was elevated in untreated PWH compared to that of ART-treated PWH or HIV-negative individuals (Table 1). However, there was no difference in CSF NfL levels between HIV-negative controls, untreated PWH, and ART-treated PWH (Table 1). CSF anti-CD4 IgG levels were not correlated with CSF NfL levels in any of the study groups (data not shown). These data revealed the link between CSF anti-CD4 IgGs and CNS abnormalities in PWH.

**TABLE 1:** Clinical characteristics of study participants^*a*^

Characteristics	HIV-negative control (*n* = 16)	HIV+, no ART (*n* = 31)	HIV+/ART+/sup (*n* = 26)	*P* values (HIV+, no ART vs HIV+, ART)
Age (yrs)	54 (37–62)^*b*^	47 (35–55)	42 (36–53)	0.49
Sex ratio (male:female)	16:0	21:10	22:4	0.37
CD4^+^ T cell counts^*c*^	799 (690–914)	250 (97–500)	595 (490–678)	<0.0001
Nadir CD4^+^ T cell counts^*c*^		215 (97–490)	280 (121–457)	0.65
Plasma HIV RNA load^*d*^		4.9 (4.3–5.5)	1.3 (1.3–1.3)	<0.0001
CSF HIV RNA load^*d*^		3.8 (2.9–5.1)	1.3 (1.3–1.3)	<0.0001
CSF WBCs	2 (1–3)	10 (1–19)	0 (0–2)	<0.0001
BBB permeability (Q-Alb)	4.7 (3.9–6.6)	6.3 (4.6–7.3)	4.9 (3.5–5.8)	0.0026
CSF NfL	369 (302–525)	410 (240–1100)	330 (228–593)	0.38

a BBB, blood-brain barrier; CSF, cerebrospinal fluid; HAD, HIV-associated dementia; NfL, neurofilament light chain (ng/L); Q-Alb, CSF/serum albumin ratio; WBCs, white blood cells (cells/mL).

b Data are given as means (interquartile range).

c CD4^+^ T cell count (cells/μL).

*^d^*HIV RNA load (log_10_ copies/mL).

### CSF levels of anti-CD4 IgGs were correlated with the degree of neuroinflammation related to myeloid cell activation in PWH *in vivo*.

HIV-induced neuroinflammation is associated with neurocognitive dysfunction (3, 9). Moreover, the activation of myeloid cells, including macrophages and microglia in CNS, may a be critical driver of neuronal injury, neuroinflammation, and HIV-associated neurocognitive disorders (HAND) (10–12). We further conducted several measurements of CSF-soluble markers related to neuroinflammation and myeloid cell activation as well as their correlations with CSF anti-CD4 IgG levels (Table 2). Notably, the levels of CSF neopterin, a biological marker of CNS inflammation and macrophage activation (13, 14), were correlated with CSF anti-CD4 IgG levels (*r* = 0.37, *P* = 0.0046). Furthermore, we found that CSF anti-CD4 IgG levels were positively correlated with several inflammatory biomarkers in CSF related to myeloid cell activation, including CSF levels of sCD14 (15) (*r* = 0.44, *P* = 0.02), IL-8 (*r* = 0.43, *P* = 0.0009), IP-10 (*r* = 0.38, *P* = 0.0035), MCP-4 (*r* = 0.30, *P* = 0.025), MIP-1α (*r* = 0.45, *P* = 0.0005), and MIP-1β (*r* = 0.32, *P* = 0.0164) (Table 2). However, CSF anti-CD4 IgG levels were not correlated with sCD163 and MCP-1 levels in CSF samples from PWH (Table 2). These results indicate the associations between elevated CSF anti-CD4 IgGs and the neuroinflammation associated with myeloid cell activation in HIV.

**TABLE 2:** Correlations between CSF anti-CD4 IgG and CSF inflammation markers in PWH^*a*^

CSF inflammation marker	Correlation coefficient (*r*)	*P* value
Neopterin	0.37	0.0046
sCD14	0.44	0.02
sCD163	0.31	0.11
MCP-1	–0.0003	0.998
IL-8	0.43	0.0009
IP-10	0.38	0.0035
MCP-4	0.30	0.025
MIP-1α	0.45	0.0005
MIP-1β	0.32	0.0164

a Nonparameteric Spearman correlation tests, correlation is significant at *P* < 0.05.

### Myeloid cell proinflammatory cytokine production in response to anti-CD4 mIgG (JF19) *in vitro*.

**FIG 3:**
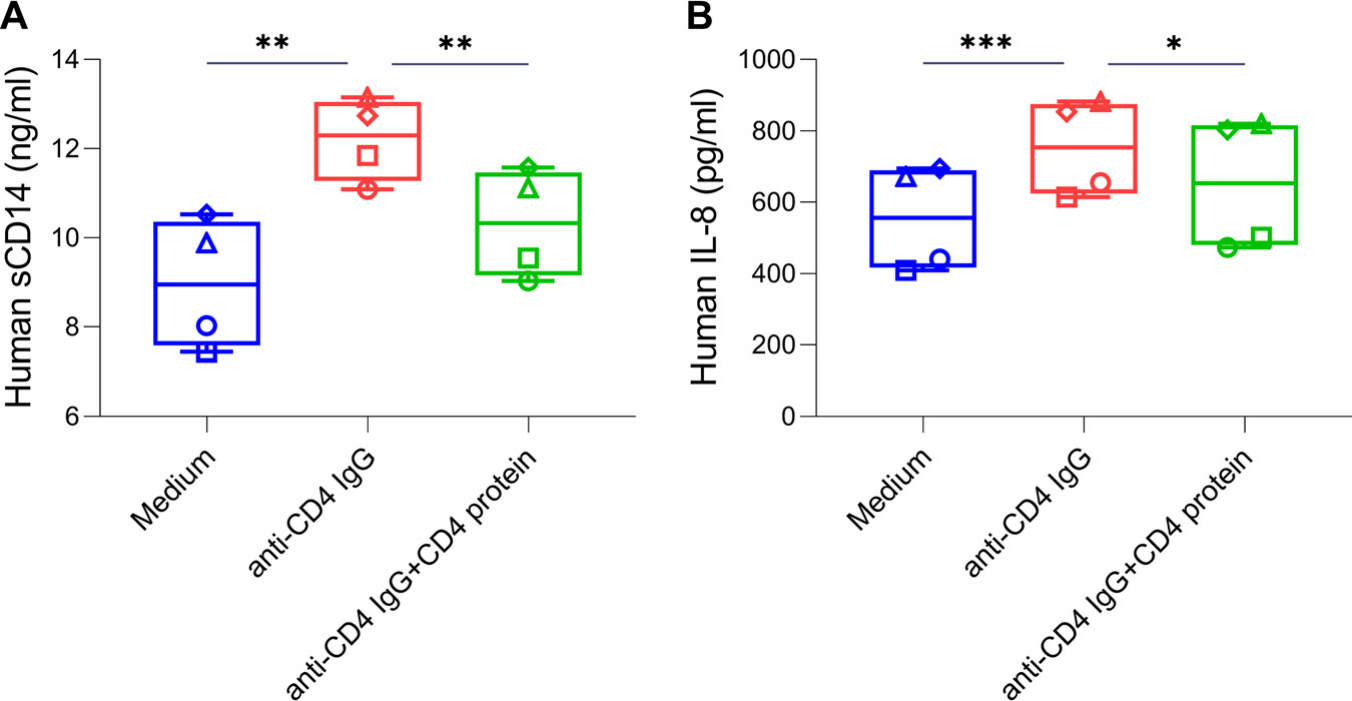
Anti-CD4 mIgG (JF19)-induced proinflammatory cytokine production in human MDM *in vitro*. MDM were generated using human monocytes from four healthy individuals. MDM were cultured with anti-CD4 mIgG (20 μg/mL) in the presence or absence of sCD4 protein (100 μg/mL) for 48 h. Levels of sCD14 (A) and IL-8 (B) were evaluated in the cell culture supernatants. The MDM from different individuals are shown as different shapes. Paired *t* test: *, *P* < 0.05; **, *P* < 0.01; ***, *P* < 0.001.

Human myeloid cells express intermediate levels of CD4 and directly respond to CD4 ligation (16, 17). To assess the potential causal relationship between CSF anti-CD4 IgG and neuroinflammation, we treated human monocyte-derived macrophages (MDM) from 4 different healthy individuals with anti-CD4 mIgG in the presence or absence of human CD4 protein for 48 h. Levels of sCD14 (Fig. 3A) and IL-8 (Fig. 3B) were increased by treatment with human anti-CD4 mIgG but were decreased in the presence of CD4 protein. In contrast, one commercial non-pathogenic human monoclonal anti-CD4 antibody, zanolimumab, and human plasma total IgG did not induce sCD14 (Fig. S2A) or IL-8 (Fig. S2B) production in human MDM. These results suggest that anti-CD4 mIgG induces sCD14 and IL-8 in MDM via CD4 binding.

## DISCUSSION

In the current study, we found elevated CSF anti-CD4 IgG levels in a subgroup of PWH despite ART. CSF levels of anti-CD4 IgG were correlated with neuroinflammation in PWH *in vivo*. In the *in vitro* study, anti-CD4 mIgG induced IL-8 and sCD14 by MDM.

Recently, autoantibodies have been found to play a role in the pathogenesis of infectious diseases, such as COVID-19 (18). However, autoantibody-mediated disease progression in HIV has been rarely reported. We were the first group to reveal that anti-CD4 IgG mediates CD4^+^ T cell death via antibody-mediated cytotoxicity (ADCC), which contributes to poor CD4^+^ T cell recovery despite suppressive ART (5, 19, 20). Further, we show high plasma anti-CD4 IgG levels in a cohort of PWH undergoing suppressive ART were associated with worse cognitive performance and brain atrophy in select regions (6). Most previous studies have investigated blood anti-CD4 IgGs in PWH, and none have been conducted on the CNS (5, 19, 21, 22). In the current study, we investigated the levels of anti-CD4 IgGs in the CSF. We found that CSF anti-CD4 IgG levels were positively correlated with neuroinflammation and the markers of myeloid cell activation, including sCD14 (associated with monocyte/macrophage activation and HAND) (23, 24; Fig. 2 and Table 2). Several factors, including neuroinflammation, HIV replication in the CNS, immune activation, and comorbidities may account for neuronal injury in HIV (25). Myeloid cells express intermediate levels of surface CD4 (17). To address the causality, we evaluated sCD14 and IL-8 production in myeloid cells in response to anti-CD4 mIgG *in vitro*; both markers are related to myeloid cell activation (26), neuroinflammation (27–29), and neuronal injury (23). We found that the patient-derived pathogenic monoclonal anti-CD4 IgG (JF19) induced sCD14 and IL-8 in MDM (Fig. 3) and that blocking of the CD4 receptor by CD4 protein had a partial inhibitory effect on the induction of inflammation. However, total IgGs isolated from human plasma and a non-pathogenic anti-CD4 antibody did not induce sCD14 and IL-8 in MDM (Fig. S2). These results suggest that JF19 may induce inflammation independent of Fc receptor.

Additionally, 20 μg/mL of JF19 used in the *in vitro* study was about 20-fold higher than CSF anti-CD4 IgG levels in some PWH *in vivo*. However, the physiologic concentration of anti-CD4 IgG in CSF may not represent the local microenvironment around myeloid cells in the CNS. The specific CD4 signaling pathway for CSF anti-CD4 IgG in myeloid cell activation and proinflammatory cytokine production in the CNS may deserve further investigation. Nonetheless, no difference in CSF NfL levels were observed in PWH compared to healthy controls, and no correlation was found between CSF anti-CD4 IgG and CSF NfL levels in PWH. However, persistent neuroinflammation can result in neuronal injury. Of note, the characteristics of a cross-sectional study may limit evaluation of the duration of neuroinflammation and its effects on neurological damage in HIV infection. Our previous studies have demonstrated ADCC activity against CD4^+^ T cells by plasma anti-CD4 IgGs from ART-nonresponsive PWH (5). Thus, we cannot exclude the possibility that increased neuroinflammation results from elevated systemic chronic inflammation which is due to anti-CD4 autoantibody-mediated poor CD4^+^ T cell recovery during ART.

A compromised BBB barrier may facilitate antibodies directly entering CSF from the blood. However, the elevated CSF anti-CD4 IgG may not only result from peripheral anti-CD4 IgG through a permeable BBB in the subgroup of PWH. (i) In Table 1, BBB permeability is increased in ART-naive patients but is normalized in ART-treated patients compared to the controls. Thus, the similar CSF anti-CD4 IgG levels in some ART-naive and ART-treated patients may not result solely from a permeable BBB. (ii) The levels of control autoantibodies anti-CD8 IgG, anti-CD4 IgM, and anti-dsDNA IgG in the CSF of PWH are much lower compared to those in the plasma. Furthermore, due to its size, IgM cannot translocate from the mother to her fetus via placenta; the absence of CSF anti-CD4 IgM suggests a non-severe compromised BBB in PWH. (iii) The ratio of CSF versus plasma levels of anti-CD4 IgG and control autoantibodies (Fig. S1) was not correlated with the degree of BBB permeability in any PWH group. Therefore, a permeable BBB in HIV cannot fully account for the elevated CSF anti-CD4 IgG in a subgroup of PWH. Nonetheless, the mechanisms of increased CSF anti-CD4 IgG in some PWH deserve further investigation.

There are several possible sources of CSF anti-CD4 IgG in some PWH. (i) The CNS infiltrated cells, including myeloid cells (intermediated surface CD4 expression) and CD4^+^ T cells, may carry the anti-CD4 IgG through surface binding. We have previously shown that anti-CD4 IgG can bind to CD4 receptor on CD4-expressing cells (19, 30). (ii) There is a germinal center-like organ in the brain in some PWH. A germinal center-like organ in local non-lymphoid tissues (e.g., kidney) has been reported in autoimmune diseases, such as systemic lupus erythematosus (SLE), and may promote local autoantibody production and tissue pathology (31). (iii) Anti-CD4 IgG-producing plasma cells infiltrate to the CNS. Plasma cells can be generated in secondary lymphoid organs, and later accumulate and persist in the inflamed non-lymphoid organs (e.g., kidney); these plasma cells enhance local concentrations of antibodies and immunocomplexes and kidney pathology in SLE (32). However, the mechanism of CNS infiltration of anti-CD4 IgG-specific plasma cells in some PWH remains unknown.

There are several limitations in this study: (i) although anti-CD4 mIgG induced sCD14 and IL-8 in MDM *in vitro*, most results shown were associations, which does not result in a cause-effect relationship; (ii) the relatively small sample size of the study may limit its power to obtain significant effects, especially in the treated PWH group with poor CD4^+^ T cell recovery; and (iii) the exact source of CSF anti-CD4 IgG in some PWH is not clear. Nonetheless, a subgroup of PWH presented with elevated CSF anti-CD4 IgG regardless of ART. This is the first study to report the associations between CSF anti-CD4 IgG levels and neuroinflammation, BBB permeability, and myeloid cell activation in CNS, suggesting a link between CNS anti-CD4 IgG and neuropathogenesis in HIV.

## MATERIALS AND METHODS

### Study subjects.

Plasma and paired CSF samples from 16 HIV-negative controls; 31 untreated, viremic PWH, including 3 subjects with HIV-associated dementia (HAD); and 26 ART-treated PWH with plasma virological suppression were evaluated in a cross-sectional study. All participants were recruited from Sahlgrenska University Hospital (Gothenburg, Sweden) and San Francisco General Hospital, University of California San Francisco (UCSF; CA, USA). This study was reviewed and approved by the local institutional review board with informed content obtained from all participants. PWH presenting with symptoms of CNS opportunistic infection or other complications were excluded. The HAD diagnosis was described in our recent publication (33). The cutoff value of the anti-CD4 IgG (ng/mL) was set at 27 based on the upper quartile of plasma levels in uninfected control individuals (5). Thus, PWH were stratified into elevated anti-CD4 IgG and normal anti-CD4 IgG subgroups for CSF and plasma samples. Additionally, the cutoff values of anti-CD4 IgM, anti-CD8 IgG, and anti-dsDNA IgG levels (mean fluorescence intensity [MFI]) were set at 504, 1,046, and 470, respectively, based on the upper quartile of corresponding plasma levels in uninfected control individuals (5).

### CSF and blood sample collection.

Lumbar CSF samples were obtained at two academic centers, as described previously (34, 35). Briefly, CSF samples were centrifuged at 400 g for 10 min to pellet cells. The supernatant was aliquoted and stored under −80°C for further analysis. Plasma samples were collected in EDTA-containing tubes, aliquoted, and similarly stored for later analysis.

### CNS measurements.

The background clinical and laboratory features of participants are summarized in Table 1. The HIV-1 RNA levels in CSF and plasma were evaluated using the Roche Amplicor version 1.5, Roche TaqMan Assay version 1 or 2 (Hoffman-La Roche Ltd., Basel, Switzerland), or Abbott RealTime HIV-1 Assay (Abbott Laboratories, Abbott Park, IL, USA) (36). The “undetectable” viral loads that were reported below the lower limit of quantitation (50 copies/mL for Amplicore and 40 copies/mL for Abbott) were standardized to a defined “floor” value of 19 copies/mL (log_10_ value of 1.28) for descriptive purposes. CSF white blood cell (WBC) counts and blood CD4^+^ and CD8^+^ T lymphocyte counts were measured using flow cytometry in local clinical laboratories. The CSF versus serum albumin quotient, an indicator of BBB permeability (37), was evaluated by a Behring Nephelometer (Behringwerke, Marburg, Germany). CSF NfL level was analyzed using a sandwich ELISA kit (UmanDiagnostics, Umeå, Sweden) in the Clinical Neurochemistry Laboratory in Gothenburg (38).

### Autoantibody measurement.

Plasma and CSF levels of autoantibodies against CD4, CD8, and double-strand DNA (dsDNA) self-antigens were conducted by Microarray and Immune Phenotyping Core Facility at the University of Texas Southwestern Medical Center, as described in our previous studies (39). Plasma samples were first treated with DNase I, then diluted with PBS at 1:50, and finally added to antigen array plates coated with autoantigens. The averaged net fluorescent intensity (MFI) of each autoantigen was normalized to internal controls, and scaling and centering were performed after log_10_ transformation according to the following formula: (x – mean[x])/sd(x). The human monoclonal anti-CD4 antibody zanolimumab (Genmab) was used to generate the standard curves.

### Soluble neuroinflammatory biomarker measurement.

Levels of soluble CD14 (sCD14) and sCD163 were assessed by an ELISA kit (R&D, Minneapolis, MN). CSF neopterin levels were analyzed using a commercially available immunoassay (BRAHMS, Hennigsdorf, Germany), with an upper value of 5.8 nmol/L of CSF (13). The following inflammatory markers, IL-8, IP-10, MCP-4, MIP-1α, and MIP-1β, were evaluated using a kit from MSD according to the manufacturer’s instruction (Meso Scale Discovery, Rockville, MD, USA). Briefly, the precoated plate was washed three times and 25 μL undiluted CSF samples were added into corresponding wells with shaking for 2 h at room temperature (RT). After washing, 25 μL of detection antibody solution was added to each well with incubation for 2 h at RT. The plate was washed again and 150 μL read buffer was added into each well. The plate was analyzed using an MSD instrument.

### Human anti-CD4 monoclonal IgG (JF19).

Human monoclonal anti-CD4 IgG (anti-CD4 mIgG) was generated from one HIV-infected, ART-treated nonresponder (clone no. JF19) with cocaine use disorder. Briefly, single IgG^+^ B cells were sorted using flow cytometry, the 10 single B cells with highest anti-CD4 IgG production were selected, B-cell receptor heavy and light chains were amplified, and monoclonal antibodies were generated following published protocol (40, 41). One pathogenic anti-CD4 mIgG was identified with ADCC activity against CD4^+^ T cells (Clonenumber JF19).

### Monocyte-derived macrophages proinflammatory cytokine production in response to anti-CD4 IgG *in vitro*.

Human primary monocytes were isolated in peripheral blood mononuclear cells (PBMCs) from healthy individuals using a Pan Monocyte Isolation Kit (Miltenyi Biotec, Bergisch Gladback, Germany). The purity ranged from 80 to 90%. The isolated monocytes were treated with 50 ng/mL M-CSF (STEMCELL, Vancouver, Canada) for 6 days. The medium was changed on day 3. At the end of day 6, the monocytes were induced into M0 MDM. Total IgG was purified from plasma of healthy controls using Protein A/G Agarose Beads (Pierce, Pittsburgh, PA). The MDM were treated with anti-CD4 monoclonal IgG (20 μg/mL), human monoclonal antibody zanolimumab (HuMax-CD4; Genmab), and human plasma total IgGs with or without human CD4 protein (100 μg/mL) for 48h. The cell culture supernatant was harvested and the concentrations of sCD14 and IL-8 in the supernatant were detected using ELISA kits (CUSABIO, Houston, TX). All of the primary cells were cultured in RPMI 1640 medium supplemented with 10% fetal bovine serum, 50 μg/mL penicillin/streptomycin, and 1 mM sodium pyruvate.

### Statistical analysis.

The data analysis and graph presentation were performed by GraphPad Prism software version 6.02 (GraphPad Software, San Diego, CA, USA). Statistical significances between groups were determined by nonparametric Mann-Whitney U tests and paired *t* tests for two-group comparisons and by one-way ANOVA and Kruskal-Wallis tests for more than two-group comparisons. Correlations between pairs of variables were evaluated by Spearman’s rank correlation test. All tests were two-tailed and *P* < 0.05 was considered to denote statistical significance.
