# Supplementary material for: Elevated Cerebrospinal Fluid Anti-CD4 Autoantibody Levels in HIV Associate with Neuroinflammation
Source: Microbiol Spectr. 2022 Jan 5;10(1):e01975-21. doi: 10.1128/spectrum.01975-21 (PMC8729763; doi:10.1128/spectrum.01975-21)
Supplement: SUPPLEMENTAL FILE 1 — Supplemental material. Download SPECTRUM01975-21_Supp_1_seq5.pdf, PDF file, 0.3 MB [file spectrum01975-21_supp_1_seq5.pdf]

Figure legends

Figure S1. Correlations between the degree of BBB permeability and the ratios of autoantibody in the CSF versus blood. Correlations between the ratio of albumin in CSF to serum and the ratio of anti-CD4 IgG (A), anti-CD4 IgM (B), anti-CD8 IgG (C), and anti-dsDNA IgG (D) in CSF versus plasma in PWH. Spearman correlation tests.

Figure S2. The non-pathogenic human monoclonal anti-CD4 antibody or human plasma total IgGs fail to induce sCD14 and IL-8 production in human MDM *in vitro*. MDM were treated with a monoclonal anti-CD4 IgG, zanolimumab or human plasma total IgG at 20 μg/ml for 48h. Levels of sCD14 (A) and IL-8 (B) were quantified in the cell culture supernatants. Summarized results in MDM from different subjects. Paired t-test.

Figure S1

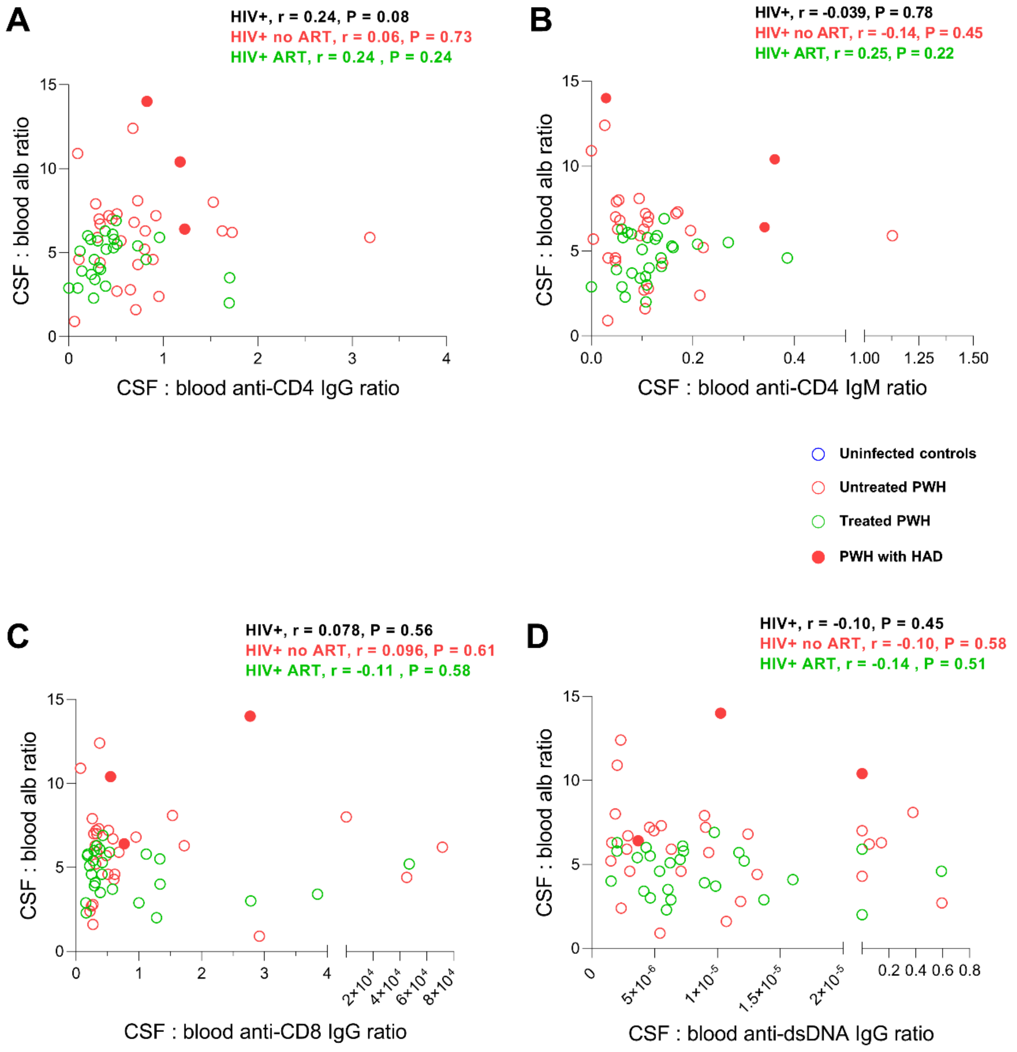
A
HIV+, r = 0.24, P = 0.08
HIV+ no ART, r = 0.06, P = 0.73
HIV+ ART, r = 0.24 , P = 0.24
CSF : blood alb ratio
CSF : blood anti-CD4 IgG ratio
B
HIV+, r = -0.039, P = 0.78
HIV+ no ART, r = -0.14, P = 0.45
HIV+ ART, r = 0.25, P = 0.22
CSF : blood alb ratio
CSF : blood anti-CD4 IgM ratio
Uninfected controls
Untreated PWH
Treated PWH
PWH with HAD
C
HIV+, r = 0.078, P = 0.56
HIV+ no ART, r = 0.096, P = 0.61
HIV+ ART, r = -0.11 , P = 0.58
CSF : blood alb ratio
CSF : blood anti-CD8 IgG ratio
D
HIV+, r = -0.10, P = 0.45
HIV+ no ART, r = -0.10, P = 0.58
HIV+ ART, r = -0.14 , P = 0.51
CSF : blood alb ratio
CSF : blood anti-dsDNA IgG ratio

Figure S2

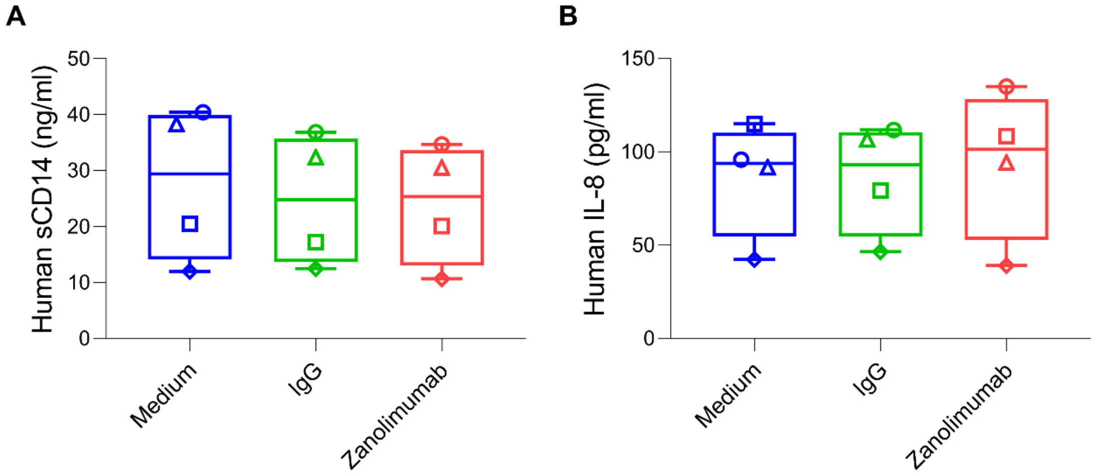

A
Human sCD14 (ng/ml)
0
10
20
30
40
50
Medium
IgG
Zanolimumab
B
Human IL-8 (pg/ml)
0
50
100
150
Medium
IgG
Zanolimumab
